# The Government and the Voluntary System

**Published:** 1922-01

**Authors:** 


### THE GOVERNMENT AND THE VOLUNTARY SYSTEM.

WE comment elsewhere on the following letter to *The Times* under date December 6, in which Sir Alfred Mond, the Minister of Health, sets forth the policy of the Government with regard to the voluntary hospitals :—

I trust that you will allow me an opportunity of replying to the letter of the Bishop of Birmingham which appeared in *The Times* on the 2nd inst. A charge of inhumanity is a grave charge to make against any public Department, but it is specially grave when it is made against the Department charged with the oversight of the physical welfare of the nation. What are the facts on which the Bishop bases his charge ? Lord Cave's Committee, which was specially appointed by my Department to investigate and report, estimated that the voluntary hospitals of Great Britain would, in the aggregate, require for maintenance during 1921 £1,000,000 more than the income expected to be available for this purpose. This estimate was based on the assumption that income and expenditure during 1921 would approximate closely to the ascertained figures for 1920, a perfectly reasonable assumption to make on the basis of such figures as were available at the time, though there is now reason to hope that the ultimate result may be less serious.

Lord Cave's Committee proposed that the whole of the estimated deficits for 1921 should be made good from an Exchequer grant. The Government took the view that it was not fair to the heavily burdened taxpayer, nor was it in the interests of the hospitals themselves, to relieve them of all the burden of expanding their revenue. The Government, therefore, decided that, except for a limited power to making emergency grants, one half of the million recommended by Lord Cave should be found from State funds, subject to the condition that the hospitals themselves, individually or collectively, should raise a corresponding amount. A first principle in the mind of the Government was that the voluntary system must be saved, and they felt that it would soon cease to be voluntary if the hospitals were not asked to co-operate in raising the new revenue required to meet post-war expenditure. If I were indeed so foolish as to wish to destroy the voluntary system, I could devise no surer way of doing it than by giving unconditional deficiency grants, with the certainty that each year voluntary effort would relax, subscriptions and money from sources other than the Treasury would fall off, the deficit to be met by the State would amount—slowly at first and then by leaps and bounds—and State control would inevitably follow.

But the Government have done more than promise a grant of £500,000. They have set up a Hospitals Commission on the lines recommended by Lord Cave, and composed as follows :—

Chairman : The Earl of Onslow. Members : The Marquess of Linlithgow, the Lord Clwyd, Sir Robert Hudson, G.B.E., Captain W. E. Elliot, M.C., M.P., Mr. D. O. Malcolm, Sir George Makins, G.C.M.G., C.B., F.R.C.S., Sir John Rose Bradford, K.C.M.G., C.B., &c., Sir Napier Burnett, K.B.E., F.R.C.S., Sir E. Cooper Perry, F.R.C.P., Mr. H. Wade Deacon, C.B.E., Dr. R. A. Bolam, O.B.E., M.D., Dr. R. C. Buist, M.D., F.R.C.P.

This Commission has already exercised its powers of making emergency grants to assist those London hospitals which were reported by King Edward's Hospital Fund to have reached the limit of their realisable assets. The Commission is establishing strong and representative hospital committees throughout the country, again in accordance with Lord Cave's recommendations. I understand that these committees will be at work in most of the more important areas before the end of the year, and I hope for the most beneficent results from their co-ordinated and combined efforts.

The " new money " required to earn a grant may be either " raised or in sight." Already I am glad to be able to say that a considerable amount of new money is in sight. The amount appropriated by the National Deposit, the Prudential and the National Amalgamated Approved Societies, and the other Approved Societies under the scheme recently set out in your columns is expected to amount to over £100,000 a year, and though this is not all net gain, since in part it will go in reduction of payments which would otherwise have been made by the patients themselves, this generous and far-sighted scheme, which has the full approval and support of the Ministry of Health, does represent a very large amount of " new money." Other important schemes, which it would be premature to discuss in detail at this juncture, for strengthening the position of the London hospitals are now being developed by the King's Fund, in consultation with the Hospitals Commission. The position is still difficult and anxious, and the Government urge strongly on the hospitals and on the community that efforts to help must not be relaxed, but encouraged and developed in all possible ways. It must, however, surely be clear to all fair-minded men that the Bishop of Birmingham does no service to the cause of the voluntary hospitals by basing charges of " inhumanity " on so flimsy a foundation. As to the wisdom of the Government's policy there may be room for legitimate difference of opinion, but reckless and unfounded charges are of no assistance whatever to the solution of a grave public problem.

In conclusion, I should wish to point out, in view of certain comments made in *The Times* on the supposed action of the Ministry of Health, that the policy above mentioned is the policy not of the Ministry of Health alone, but of the Government, and it is a policy which was formally submitted to and approved by the House of Commons. It is beside the mark to urge that more money should have been found for the hospitals by the sacrifice of other services entrusted to the Ministry. These services have a value of their own, and it is a matter of opinion how far they should be restricted. But it should be realised that they have been gradually built up in partnership with the local authorities of the country. Capital commitments have been undertaken and heavy obligations incurred on both sides, and money cannot be diverted to other objects by the mere waving of a magician's wand. What has been done for the hospitals should be, in my judgment, adequate to save the voluntary system if the supporters of that system themselves help in the full spirit of their splendid tradition and if the community does its full share. I am confident that this will be done, that the system will withstand the present strain and, being established on a stronger foundation, will continue to keep the hospitals of this country in the forefront of progress. In this great task all the sympathy and all the help which is within the power of the Ministry of Health will be as freely at their disposal as it has been throughout the crisis.

